# Yeast Bloodstream Infections in the COVID-19 Patient: A Multicenter Italian Study (FiCoV Study)

**DOI:** 10.3390/jof9020277

**Published:** 2023-02-20

**Authors:** Anna Prigitano, Elisabetta Blasi, Maria Calabrò, Caterina Cavanna, Maria Cornetta, Claudio Farina, Anna Grancini, Patrizia Innocenti, Giuliana Lo Cascio, Lucia Nicola, Laura Trovato, Massimo Cogliati, Maria Carmela Esposto, Anna Maria Tortorano, Luisa Romanò, on behalf of the FiCoV Study Group

**Affiliations:** 1 Department of Biomedical Sciences for Health, Università degli Studi di Milano, 20133 Milan, Italy; 2 Laboratory of Microbiology, AOU-Policlinic/CHIMOMO, University of Modena and Reggio Emilia, 41121 Modena, Italy; 3 Clinical Microbiology and Virology Laboratory, Humanitas Research Hospital, 20089 Rozzano, Italy; 4 Microbiology and Virology Department, Fondazione IRCCS Policlinico San Matteo, 27100 Pavia, Italy; 5 Operative Unit 1—Clinical Pathology, Department of Pathology and Laboratory Medicine, IRCCS Policlinico San Donato, 20097 San Donato Milanese, Italy; 6 Microbiology and Virology Laboratory, ASST “Papa Giovanni XXIII”, 24100 Bergamo, Italy; 7 U.O.S. Microbiology—Analysis Laboratory, IRCCS Foundation, Cà Granda Ospedale Maggiore Policlinico, 20122 Milan, Italy; 8 Laboratorio Aziendale di Microbiologia e Virologia di Bolzano, Comprensorio Sanitario di Bolzano, 39100 Bolzano, Italy; 9 Dipartimento di Patologia Clinica-Unità Operativa di Microbiologia e Virologia-AUSL Piacenza, 29121 Piacenza, Italy; 10 ASST Melegnano e Martesana, Laboratorio Microbiologia PO Cernusco s/N, 20063 Cernusco sul Naviglio, Italy; 11 U.O.C. Laboratory Analysis Unit, A.O.U. “Policlinico-San Marco”, 95125 Catania, Italy; 12 Department of Biomedical and Biotechnological Sciences, University of Catania, 95124 Catania, Italy

**Keywords:** COVID-19, candidemia, fungemia, invasive fungal infections, antifungal resistance

## Abstract

Fungemia is a co-infection contributing to the worsening of the critically ill COVID-19 patient. The multicenter Italian observational study FiCoV aims to estimate the frequency of yeast bloodstream infections (BSIs), to describe the factors associated with yeast BSIs in COVID-19 patients hospitalized in 10 hospitals, and to analyze the antifungal susceptibility profiles of the yeasts isolated from blood cultures. The study included all hospitalized adult COVID-19 patients with a yeast BSI; anonymous data was collected from each patient and data about antifungal susceptibility was collected. Yeast BSI occurred in 1.06% of patients, from 0.14% to 3.39% among the 10 participating centers. Patients were mainly admitted to intensive or sub-intensive care units (68.6%), over 60 years of age (73%), with a mean and median time from the hospitalization to fungemia of 29 and 22 days, respectively. Regarding risk factors for fungemia, most patients received corticosteroid therapy during hospitalization (61.8%) and had a comorbidity (25.3% diabetes, 11.5% chronic respiratory disorder, 9.5% cancer, 6% haematological malignancies, 1.4% organ transplantation). Antifungal therapy was administered to 75.6% of patients, mostly echinocandins (64.5%). The fatality rate observed in COVID-19 patients with yeast BSI was significantly higher than that of COVID-19 patients without yeast BSI (45.5% versus 30.5%). *Candida parapsilosis* (49.8%) and *C. albicans* (35.2%) were the most fungal species isolated; 72% of *C. parapsilosis* strains were fluconazole-resistant (range 0–93.2% among the centers). The FiCoV study highlights a high prevalence of *Candida* BSIs in critically ill COVID-19 patients, especially hospitalized in an intensive care unit, a high fatality rate associated with the fungal co-infection, and the worrying spread of azole-resistant *C. parapsilosis.*

## 1. Introduction

The most serious clinical forms of Coronavirus Disease of 2019 (COVID-19) are characterized by severe acute respiratory distress syndrome (ARDS), cytokine storm, and death. Roles in the worsening of clinical conditions in critical COVID-19 patients can be played by bacteria, yeast, and mold co-infections [1]. Risk factors associated with a secondary infection are epithelial barrier damage, the widespread use of antibiotics, an immune system dysregulation, a prolonged hospitalization, and the admission to an intensive care unit (ICU) [2,3,4]. Fungemia is known to occur as a secondary infection in critically ill patients admitted to ICU [5] and in COVID-19 patients [6,7]. In particular, candidemia is a common nosocomial bloodstream infection in the critically ill patient, ranking between the third and fifth most commonly isolated microorganisms in ICU-acquired bloodstream infections (BSIs) [8], and characterized by a high crude mortality (25–50%) even in patients undergoing treatment [9]. Harboring a strain resistant to antifungals makes the management of the COVID-19 patient with a fungal infection more difficult. Indeed, antifungal resistance is a global emergency in nosocomial environments. Recently, the emergence of multi-resistance in *Candida auris* [10] and fluconazole resistance in *Candida parapsilosis* [11] has been reported worldwide.

With the aim to evaluate the fungal infections in hospitalized COVID-19 patients, including fungal bloodstream infections, we conducted an observational multicenter study in Italy named “Fungal Infections in COVID-19 Patients—FiCoV Study”. The primary aims of the FiCoV Study were to estimate the frequency of yeast bloodstream infections (BSIs), to describe the risk factors associated with the presence of fungemia in COVID-19 patients, and to analyze the antifungal susceptibility profiles of the yeasts isolated from blood cultures. The secondary aim was to calculate the case fatality rate in COVID-19 patients with fungemia compared to that in COVID-19 patients without fungemia.

## 2. Materials and Methods

### 2.1. Study Design

We conducted an observational study (FiCoV Study) in which data from February to May 2020 was collected retrospectively and data from June 2020 to June 2021 was collected prospectively. The present multicenter study involved 10 hospitals (named from H1 to H10) located in Northern (Lombardia *n* = 6, Emilia Romagna *n* = 2, Trentino Alto Adige *n* = 1) and Southern Italy (Sicilia *n* = 1). All the participant centers were tertiary hospitals with a mean number of 725 beds, range 118–1108. During the study period, all participant centers had at least one COVID-19 dedicated ICU and sub-ICU (for 9 centers); in addition, most beds of medical wards were converted for COVID-19 patients, with a variable number of beds according to the different epidemic waves.

The study was approved by the Ethical Committees of the University of Milan (Coordinator Center) and those of the participating hospitals. The study included all hospitalized adult (≥18 years) COVID-19 patients with a yeast BSI, either already present upon the patient’s admission or developed during hospitalization. Additionally, in our study, a patient with at least one blood culture positive for yeasts was listed as a yeast BSI case.

Anonymous data were collected for each patient concerning demographic characteristics, hospitalization ward, underlying comorbidities (hematological malignancies, oncological diseases, diabetes, and chronic respiratory diseases), the use of corticosteroids before and during COVID-19 infection, antifungal treatment, and the patient’s outcome at the discharge date from the hospital. In addition, we collected data regarding the number of positive blood cultures and the date in which they were collected, fungal species identification, culture of intravascular lines, and antifungal susceptibility. Each participating hospital also provided the total number of COVID-19 patients hospitalized and the number of deaths due to COVID-19 during the study period. All data collected were sent, in a dedicated form, to the Coordinator Center (Medical Mycology Laboratory, Department of Biomedical Sciences for Health of Università degli Studi di Milano).

### 2.2. Isolates Identification and Susceptibility Testing

Blood cultures were performed according to the clinician’s evaluation, on the basis of clinical signs compatible with a sepsis or a suspected catheter-related infection. Blood cultures were processed using BacT/Alert (BioMérieux, Marcy l’Etoile, France) in four participating hospitals and by BD-Bactec (Becton Dickinson, Franklin Lakes, NJ, USA) in the other six, using classic aerobic bottles.

The identification of the isolates was performed in the participating hospital laboratories using Vitek 2 Yeast cards BioMerieux (1 laboratory), Matrix-assisted laser desorption/ionization time of flight mass spectrometry (MALDI-TOF-MS, Bruker Daltonics, Bremen, Germany; 5 laboratories), and Vitek MS BioMérieux (4 laboratories).

Antifungal susceptibility was performed with Sensititre YeastOne (SYO, Thermo Scientific Trek Diagnostic Systems, East Grinstead, UK) in all centers but one, that used Vitek 2 (BioMérieux). Minimal inhibitory concentration (MIC) values obtained by SYO were interpreted according to Clinical Laboratory and Standards Institute (CLSI) species-specific breakpoints (BPs) or, when breakpoints lacked, according to the SYO epidemiological cutoff values (ECV) to distinguish between wild type and non-wild type isolates [12,13]. MICs obtained with Vitek 2 were interpreted according to the European Committee on Antimicrobial Susceptibility Testing [14] BPs, and, in presence of resistance, confirmed by SYO.

### 2.3. Statistical Analysis

The descriptive analysis of data was performed using distributions of frequency, relative frequency, percentage, and measures of central tendency. The Chi-square test was used to compare categorical variables. Only statistically significant values with *p* < 0.05 were reported. To determine the relationship between yeast BSI frequency and risk factor, a Pearson test was performed.

## 3. Results

Among the total of 27,981 COVID-19 patients admitted to the 10 Italian hospitals involved in the FiCoV Study during the 17-month study period, yeast BSI occurred in 296 (1.06%) patients; specifically, in 106 patients in the period of February–May 2020 and in 190 patients between June 2020 and June 2021. The frequency of the yeast BSI among the different centers ranged from 0.14% to 3.39%, with a single center (H2) having a significantly (*p* < 0.001) higher frequency (Table 1).

As reported in Table 2, most of the 296 COVID-19 patients with yeast BSI were males (77.7%, 230/296), and aged over 60 years old (73%, 216/296).

The analysis of the risk factors for the development of a fungal infection showed that 183 (61.8%) patients received corticosteroids during hospitalization as treatment for COVID-19 (*n* = 166) or for other underlying conditions (*n* = 17). Other risk factors included diabetes (25.3%; 75/296), a chronic respiratory disorder (11.5%; 34/296), cancer (9.5%; 28/296), hematological malignancy (6%; 18/296), and organ transplant (1.4%, 4). One hundred and five patients (35.5%) had ≥2 co-morbidities such as hypertension (28.7%, 85/296), cardiopathy (13.5%; 40/296), obesity (11.1%; 33/296), and dyslipidemia (5.7%; 17/296) (Table 2). A central venous catheter (CVC) was present in 81.4% of COVID-19 patients with yeast BSI. Statistical analysis did not reveal any correlation between the frequency of the risk factors examined and the frequency of fungal infection among the different centers.

Of the 296 COVID-19 patients with a yeast BSI, most (*n* = 203; 68.6%) were admitted to an ICU (182/296, 61.5%) or sub-ICU (21/296, 7.1%), while the remaining 92 patients (31.1%) were admitted to other wards that were converted to infectious disease wards for COVID-19 cases during the pandemic period (*p* < 0.001); finally, one (0.3%) patient remained in the emergency room until his death. The median period of hospitalization was 46 days (range 2–467 days). A prolonged hospitalization (≥14 days) was reported for 89.2% (232/260) of the patients.

Data regarding the occurrence of the BSI were available for 268 of the 296 COVID-19 patients with yeast BSI. The mean and median time from the hospitalization for COVID-19 to the occurrence of BSI was 29 days and 22 days (range 0–342 days), respectively. Furthermore, 12 (4.5%) patients had fungemia at the time of admission and 7 (2.6%) had been diagnosed during the first 48 h after admission, while a significant number (249, 93%; *p* < 0.001) of patients developed a positive blood culture in the next days, namely 52% between the 11th and 30th day.

An antifungal therapy was administered to a total of 192 out of 254 (75.6%) patients; however, complete information was available only for 156 patients. Initial antifungal therapy was an echinocandin in 57.1% (89/156) of the patients, fluconazole in 24.4% (38/156), other azoles in 10.9% (17/156), and amphotericin B in 1.9% (3/156) of the patients. Six (3.8%) patients received combined therapy with an echinocandin and an azole, while 3 (1.9%) patients received an echinocandin combined with amphotericin B (Table 2). During treatment, initial therapy was switched to a drug of the same antifungal class (7 patients) or to one of a different class (38 patients).

During the study period, data on mortality was available for 288 patients, out of which 131 died (45.5%). The fatality rate was similar between COVID-19 patients hospitalized in ICU/sub-ICU or in medical wards (45.8% vs. 44.7%, *p* = 0.864). On the contrary, the fatality rate was significantly higher in COVID-19 patients with yeast BSI compared to that observed in COVID-19 patients without yeast BSI (45.5% vs. 30.5%; *p* < 0.001). Among COVID-19 patients with yeast BS co-infection, the median interval between the first blood culture positive for yeast and death was 11 days (range 0–199 days); most patients (61%, 80/131) died within 15 days from the first positive blood culture (Table 2). In addition, 8 of the 17 patients (47.1%) who died prematurely (within 2 days after fungal isolation) did not receive antifungal therapy and 67 patients (67/131; 51.1%) died despite antifungal therapy.

Regarding the identification of isolates, the most common fungal species isolated were *Candida parapsilosis* (49.8%; 160/321), *Candida albicans* (35.2%; 113/321), *Candida glabrata* (10%; 32/321), *Candida tropicalis* (2.8%; 9/321), *Candida lusitaniae* (0.6%; 2/321), and *Candida metapsilosis* (0.6%; 2/321). Moreover, *Saccharomyces cerevisiae* was isolated from three patients (0.9%; 3/321) (Table 3).

A different species distribution was observed among the different centers. Specifically, in the H2 hospital, the frequency of *C. parapsilosis* was significantly higher than in other centers (75.4%, *p* < 0.05); *C. albicans* was significantly more isolated (*p* < 0.02) in H1 hospital than in H2, H6, and H8; and the isolation of *C. glabrata* was significantly greater in H10, H2, H3, H6, and H9 (*p* < 0.05) than in the other hospitals.

The highest fatality rate was observed for COVID-19 patients with *C. glabrata* (50%), *C. tropicalis* (50%), *C. albicans* (48.5%), and *C. parapsilosis* (39.9%). 

Detailed in vitro susceptibility results are reported in supplemental materials (Table S1). The analysis of susceptibility results showed that 72.6% (106/146) of *C. parapsilosis* isolates were resistant to fluconazole, with a resistance rate among the participating centers ranging from 0 (3 centers) to 93.2% (1 center). In particular, H2 center showed a higher rate of resistance than other participant centers (*p* < 0.0001), except H8 and H10 centers. In addition, 31.5% (46/146) of *C. parapsilosis* strains showed a multiple resistance to fluconazole and voriconazole. Among the *C. glabrata* strains, 42.8% (12/28) were resistant to itraconazole, 10.7% (3/28) to fluconazole, and two strains were resistant to both itraconazole and fluconazole. One of the 9 (11.1%) *C. tropicalis* tested strains were resistant to fluconazole, itraconazole, and voriconazole, but susceptible to echinocandins and amphotericin B. Only 3.8% (4/104) of *C. albicans* isolates showed fluconazole resistance, similarly to posaconazole (4%) and itraconazole (2.4%); two strains showed a multiple resistance, one to the four tested azoles, and another to fluconazole and itraconazole. Furthermore, 3.5% of *C. albicans* strains showed resistance to echinocandins—both anidulafungin and micafungin—not observed in other species. A 5-fluorocytosine resistance was observed only in two out of five *C. tropicalis* tested isolates (40%). All species showed low amphotericin B MIC values (range 0.12–1 mg/L). For the species less frequently isolated (*C. lusitaniae, C. metapsilosis*, and *S. cerevisiae*) and in the absence of species-specific BPs, MIC values are reported in Table 4.

Finally, the case fatality rate of COVID-19 patients harboring a resistant fungal strain was comparable to that of patients harboring a susceptible strain (45% vs. 43.1%; *p* = 0.758) and no significant differences were observed among different fungal species.

## 4. Discussion

A recent review on fungemia in COVID-19 patients highlights an average incidence of fungemia of 3.8% with a wide difference between the centers (range from 0.4% to 44.6%) [15].

The strength of the present study was the possibility of conducting a multicenter study, which involved 10 hospitals located in different parts of Italy in order to study fungemia on approximately 28,000 patients hospitalized for COVID-19. The observed mean frequency (1.06%) of yeast BSI was lower than that reported by other authors [6,15,16], confirming the difference between hospitals (from 0.14% to 3.39%).

During the pandemic, a significant increase in *Candida* bloodstream infections has been observed in COVID-19 patients compared to patients without COVID-19 [17,18,19]. This increase was associated with different factors, such as dysregulation of the immune system [20,21], a longer hospitalization period, admission to ICU [3], and prolonged use of corticosteroids [19]. As reported by other authors [7,22], we observed that corticosteroid treatment (56%), as well as diabetes (25.3%), were frequent in patients who developed fungemia. However, because the data has not been compared to a control group of COVID-19 patients without yeast BSI, we could not establish an association between these factors and yeast BSI. The role of corticosteroids as a risk factor for fungemia is debated. Even if corticosteroids have an immunosuppressive effect, recent studies reported that the administration of corticosteroids is not an independent factor for the candidemia [23,24]. This is probably because their use leads to a clinical improvement of the critical conditions in COVID-19 patients, reducing the length of the ICU stay and decreasing the risk of the patients’ exposure to invasive medical procedures predisposing to development of infections.

Comorbidities that are frequently considered risk factors for fungemia, such as cancer, hematological malignancy, or surgery, were not frequent in our study, as observed in other studies [24,25]. Instead, the presence of two or more comorbidities, observed in more than 35% of our patients with yeast BSI, seems to represent an increased risk for candidemia [24,26]. Another important factor associated with fungal co-infection is the presence of a CVC insertion [27] as observed in 81.4% of FiCoV study patients during their hospitalization.

The antifungal treatment of FiCoV patients with echinocandins (64.5%), particularly caspofungin, was confirmed as the preferred treatment, in line with other studies [7,16,28].

During the FiCoV study, the fatality rate (45.5%) observed in COVID-19 patients with fungemia was significantly higher than that of COVID-19 patients without fungemia (30.5%). The fatality rate, although high, was still lower than the 74.8% reported by a review analysis of 25 international studies [15]. 

In the present study, *C. parapsilosis* was the yeast most frequently isolated (49.8% of the cases), followed by *C. albicans* and *C. glabrata* (35% and 10% respectively). The high frequency of *C. parapsilosis* was in contrast with the data of the literature reporting *C. albicans* and *C. glabrata* as the prevalent species in COVID-19 patients [6,16,17] but also in other patients in the pre-COVID era [29].

Of note, the *C. parapsilosis* prevalence varied greatly among the centers (range 0–75.4%). The local fungal etiology could be the basis of the particular result obtained in this study, especially in regards to the H2 center in which a significant prevalence of *C. parapsilosis* in the BSIs had already been observed and studied in the pre-COVID era (data not yet published). The highest prevalence of resistance was also observed in the same center. It is well known that *C. parapsilosis* candidemia is associated with an exogenous acquisition, thus the extensive use of a CVC in COVID-19 patients, in addition to the pandemic emergency and the pressure on the ICUs, may have contributed to an incorrect management of the catheter favoring *C. parapsilosis* candidemia. In recent years, outbreaks of fluconazole-resistant *C. parapsilosis* infections have been described worldwide and the resistant isolates appear to be more likely to spread over a long period of time than susceptible ones, and to be more frequently associated with invasive infections [11,30]. Fluconazole resistance in *C. parapsilosis* may emerge as a consequence of the pharmacological pressure of fluconazole treatment or prophylaxis, and possible patient-to-patient transmission in the hospital setting [31]. In addition, the ability to form tenacious biofilms on vascular catheters and other medically implanted devices is responsible for azole resistance [9]. In the FiCoV study, parallel with the increase of *C. parapsilosis* bloodstream infections, an increase in fluconazole-resistant *C. parapsilosis* isolates has been observed (72.6%), higher than reported in previous Italian studies [32]. The higher presence and circulation of two clusters of azole-resistant *C. parapsilosis* isolates in one center had been known in the pre-COVID-19 era (data not yet published). However, the conditions created during the pandemic have probably amplified this phenomenon and spread these strains.

In addition, compared to the data in the pre-COVID-19 era, in this study, we have observed an increase in cross-resistance [32,33].

The study has some limitations due to the lack of data, especially those related to retrospectively enrolled patients, and to the non-generalizability of the results given the local fungal epidemiology of the study centers.

## 5. Conclusions

This large study performed in Italy highlights the high prevalence of *Candida* BSIs in critically ill COVID-19 patients, especially in those hospitalized in ICU, confirming what has been reported since the beginning of the pandemic and highlighting the high fatality rate associated with fungal co-infection. Further studies will be needed to better understand the risk factors for development of fungemia, such as the role of corticosteroids, to assist clinicians in improving the management of critically ill COVID-19 patients and avoiding the onset of this serious complication. Moreover, the worrying spread of azole-resistant *C. parapsilosis* isolates should induce clinicians, in collaboration with microbiology laboratories, to devote particular attention to the epidemiological situation of their center and to implement antifungal susceptibility testing.

## Figures and Tables

**Table 1:** Frequency of yeast BSI in the COVID-19 patients hospitalized during the study period (February 2020–June 2021) in the 10 participating hospitals.

Hospital	N. of COVID-19 Patients	
	Total	with Yeast BSI (%)
H1	4644	30 (0.65)
H2	3070	104 (3.39)
H3	5288	41 (0.78)
H4	3341	35 (1.05)
H5	1478	14 (0.95)
H6	2374	21 (0.88)
H7	2798	19 (0.68)
H8	1462	9 (0.62)
H9	1433	20 (1.40)
H10	2093	3 (0.14)
Total	27,981	296 (1.06)

**Table 2:** Main characteristics of 296 COVID-19 patients with yeast BSI.

Characteristics	N. (%)
Gender (Male)	230 (77.7%)
Age group (years):	
18–30	3 (1%)
31–40	4 (1.4%)
41–50	22 (7.4%)
51–60	51 (17.2%)
61–70	96 (32.4%)
71–80	89 (30%)
≥81	31 (10.5%)
ICU patients	182 (61.5%)
Sub-ICU patients	21 (7.1%)
Presence of central venous catheter	241 (81.4%)
Risk factors for fungal infection:	
Corticosteroid therapy	183 (61.8%)
Diabetes	75 (25.3%)
Respiratory chronic disorder	34 (11.5%)
Solid cancer	28 (9.5%)
Haematological malignancy	18 (6%)
Organ transplant	4 (1.4%)
Co-morbidities:	
Hypertension	85 (28.7)
Cardiopathy	40 (13.5)
Obesity	33 (11.1)
Dyslipidemia	17 (5.7)
N. of patients treated with antifungal drugs	192 (64.9%)
N. of patients with initial antifungal therapy with *:	
Caspofungin	68 (43.6%)
Fluconazole	38 (24.3%)
Anidulafungin	21 (13.5%)
Voriconazole	13 (8.3%)
Isavuconazole	1 (0.6%)
Amphotericin B	3 (1.9%)
Itraconazole	3 (1.9%)
Echinocandin + azole	6 (3.8%)
Echinocandin + amphotericin B Case fatality rate	3 (1.9%) 131 (45.5%)
Death at (day after fungal isolation):	
0–5	38 (31.1%)
6–10	24 (18%)
11–15	18 (13.7%)
16–20	12 (9%)
21–30	12 (9%)
31–40	5 (3.2%)
41–50	9 (6.5%)
51–60	3 (2.5%)
≥61	10 (7.4%)

* no information on antifungal drug was available for 36 patients.

**Table 3:** Species distribution and antifungal resistance rate in the 10 participating hospitals (H1–H10).

	H1	H2	H3	H4	H5	H6	H7	H8	H9	H10	Total
N. patients with fungemia	30	104	41	35	14	21	19	9	20	3	296
Species isolates $:											
*C. albicans*	20 (64.5%)	14 (11.7%)	19 (44.2%)	25 (58.1%)	6 (42.9%)	6 (28.6%)	10 52.6%)	2 (22.2%)	10 (50%)	1 (33.3%)	113 (35.2%)
*C. glabrata*	5 (16.1%)	11 (9.3%)	2 (4.7%)	1 (2.3%)	2 (14.3%)	1 (4.8%)	4 (21%)	1 (11.1%)	3 (15%)	2 (66.6%)	32 (10%)
*C. parapsilosis*	4 (12.9%)	89 (75.4%)	20 (46.5%)	14 (32.6%)	5 (35.7%)	14 (66.6%)	5 (26.3%)	3 (33.3%)	6 (30%)	-	160 (49.8%)
*C. tropicalis*	2 (6.5%)	3 (2.5%)	1 (2.3%)	1 (2.3%)	1 (7.1%)	-	-	-	1 (5%)	-	9 (2.8%)
*C. metapsilosis*	-	1 (0.8%)	1 (2.3%)	-	-	-	-	-	-	-	2 (0.6%)
*C. lusitanae*	-	-	-	2 (4.6%)	-	-	-	-	-	-	2 (0.6%)
*S. cerevisiae*	-	-	-	-	-	-	-	3 (33.3%)	-	-	3 (0.9%)
Total	31	118	43	43	14	21	19	9	20	3	321
Resistance to ≥1 antifungals	4/31 (12.9%)	87/118 (73.7%)	14/43 (32.6%)	8/43 (18.6%)	2/14 (14.3%)	NA	2/19 (10.5%)	3/6 (50%)	7/20 (35%)	1/3 (33.3%)	128 (43.1%)
*C. parapsilosis* fluconazole resistant	1 (25%)	83 (93.2%)	14 (70%)	4 (28.6%)	0	NA	0	2 (66.6%)	2 (33.3%)	-	106 (72.6%)

NA = antifungal susceptibility results are not available. $ some patients had a double infection.

**Table 4:** MIC_90_, MIC range, number of resistant isolates according to CLSI species-specific breakpoints (R CLSI) or non-wild type (non-WT) isolates.

		*C. albicans*	*C. glabrata*	*C. parapsilosis*	*C. tropicalis*	*C. lusitanae*	*C. metapsilosis*	*Saccharomyces*
FLUCONAZOLE	N. tested	104	28	146	9	2	2	3
	MIC_90_	1	32	128	4	0.5	2	0.25
	MIC range	0.12–256	0.5–128	0.12–256	0.5–128	0.25–0.5	1–2	0.125–0.25
	R CLSI	4 (3.8%)	3 (10.7%)	106 (72.6%)	1 (11.1%)	n.a.	n.a.	n.a.
ITRACONAZOLE	N. tested	84	28	125	9	2	1	3
	MIC_90_	0.12	1	0.25	1	0.12	0.06	16
	MIC range	0.015–16	0.12–1	0.015–0.25	0.03–1	0.06–0.12		1–16
	R CLSI	2 (2.4%)	12 (42.8%)	0	1 (11.1%)	n.a.	n.a.	n.a.
POSACONAZOLE	N. tested	74	26	120	7	2	1	3
	MIC_90_	0.06	2	0.12	0.25	0.06	0.015	2
	MIC range	0.015–8	0.25–4	0.008–0.25	0.03–0.25	0.03–0.06		1–2
	R CLSI	n.a.	n.a.	n.a.	n.a.	n.a.	n.a.	n.a.
	Non-WT	3 (4%)	0	0	0			
VORICONAZOLE	N. tested	105	27	145	9	2	2	3
	MIC_90_	0.12	1	1	0.25	0.008	0.12	0.5
	MIC range	0.008–8	0.12–1	0.008–2	0.015–1	0.008–0.008	0.015–0.12	0.25–0.5
	R CLSI	1 (0.9%)	n.a.	46 (31.7%)	1 (11.1%)	n.a.	n.a.	n.a.
	Non-WT	-	0	-	-	-	-	-
ANIDULAFUNGIN	N. tested	86	28	127	8	2	1	2
	MIC_90_	0.12	0.06	2	0.12	0.25	0.25	0.06
	MIC range	0.015–2	0.015–0.06	0.015–2	0.015–0.25	0.25–0.25		0.015–0.06
	R CLSI	3 (3.5%)	0	0	0	n.a.	n.a.	n.a.
CASPOFUNGIN	N. tested	105	29	145	9	2	2	3
	MIC_90_	0.12	0.25	1	0.12	0.5	0.5	0.06
	MIC range	0.008–0.5	0.03–0.25	0.03–2	0.03–0.25	0.25–0.5	0.25–0.5	0.03–0.06
	R CLSI	0	0	0	0	n.a.	n.a.	n.a.
MICAFUNGIN	No. tested	85	31	146	9	2	2	3
	MIC_90_	0.015	0.03	2	0.06	0.06	0.5	0.125
	MIC range	0.008–1	0.015–0.03	0.008–2	0.015–0.03	0.06–0.06	0.125–0.5	0.06–0.125
	R CLSI	3 (3.5%)	0	0	0	n.a.	n.a.	n.a.
5-FLUOROCYTOSINE	No. tested	72	22	69	5	2	1	3
	MIC_90_	0.12	8	0.25	64	2	0.5	16
	MIC range	0.06–1	0.06–8	0.06–0.5	0.5–64	0.06–2		0.06–16
	R CLSI	0	0	0	2 (40%)	n.a.	n.a.	n.a.
AMPHOTERICIN B	No. tested	95	28	140	8	2	2	3
	MIC_90_	1	1	1	1	0.5	1	0.25
	MIC range	0.12–1	0.12–1	0.12–1	0.25–1	0.25–0.5	0.25–1	0.125–0.25
	R CLSI	n.a.	n.a.	n.a.	n.a.	n.a.	n.a.	n.a.
	Non-WT	0	0	0	0	-	-	-

n.a. = not applicable.

## Data Availability

Data sharing not applicable.

## Supplementary Materials

The following supporting information can be downloaded at: https://www.mdpi.com/article/10.3390/jof9020277/s1, Table S1: In vitro susceptibilities of 299 bloodstream isolates.

## References

[1] Russell C.D., Fairfield C.J., Drake T.M., Turtle L., Seaton R.A., Wootton D.G., Sigfrid L., Harrison E.M., Docherty A.B., de Silva T.I. (2021). Co-infections, secondary infections, and antimicrobial use in patients hospitalised with COVID-19 during the first pandemic wave from the ISARIC WHO CCP-UK study: A multicentre, prospective cohort study. Lancet Microbe.

[2] Cevik M., Kuppalli K., Kindrachuk J., Peiris M. (2020). Virology, transmission, and pathogenesis of SARS-CoV-2. BMJ.

[3] Ripa M., Galli L., Poli A., Oltolini C., Spagnuolo V., Mastrangelo A., Muccini C., Monti G., De Luca G., Landoni G. (2021). Secondary infections in patients hospitalized with COVID-19: Incidence and predictive factors. Clin. Microbiol. Infect..

[4] Diao B., Wang C., Tan Y., Chen X., Liu Y., Ning L., Chen L., Li M., Liu Y., Wang G. (2020). Reduction and functional exhaustion of T Cells in patients with Coronavirus Disease 2019 (COVID-19). Front. Immunol..

[5] Chow J.K., Golan Y., Ruthazer R., Karchmer A.W., Carmeli Y., Lichtenberg D.A., Chawla V., Young J.A., Hadley S. (2008). Risk factors for *albicans* and non-*albicans* candidaemia in the intensive care unit. Crit. Care Med..

[6] Seagle E.E., Jackson B.R., Lockhart S.R., Georgacopoulos O., Nunnally N.S., Roland J., Barter D.M., Johnston H.L., Czaja C.A., Kayalioglu H. (2022). The landscape of candidaemia during the COVID-19 pandemic. Clin. Infect. Dis..

[7] Kayaaslan B., Eser F., Kaya Kalem A., Bilgic Z., Asilturk D., Hasanoglu I., Ayhan M., Tezer Tekce Y., Erdem D., Turan S. (2021). Characteristics of candidaemia in COVID-19 patients; Increased incidence, earlier occurrence, and higher mortality rates compared to non-COVID-19 patients. Mycoses.

[8] Suetens C., Morales I., Savey A., Palomar M., Hiesmayr M., Lepape A., Gastmeier P., Schmit J., Valinteliene R., Fabry J. (2007). European surveillance of ICU-acquired infections (HELICS-ICU): Methods and main results. J. Hosp. Infect..

[9] Tortorano A.M., Prigitano A., Morroni G., Brescini L., Barchiesi F. (2021). Candidemia: Evolution of drug resistance and novel therapeutic approaches. Infect. Drug Resist..

[10] Chowdhary A., Sharma C., Meis J.F. (2017). *Candida auris*: A rapidly emerging cause of hospital-acquired multidrug-resistant fungal infections globally. PLoS Pathog..

[11] Mesini A., Mikulska M., Giacobbe D.R., Del Puente F., Gandolfo N., Codda G., Orsi A., Tassinari F., Beltramini S., Marchese A. (2020). Changing epidemiology of candidaemia: Increase in fluconazole-resistant *Candida parapsilosis*. Mycoses.

[12] Clinical and Laboratory Standards Institute (2008). Reference Method for Broth Dilution Antifungal Susceptibility Testing of Yeasts; Approved Standard, 3rd ed., Wayne, PA: CLSI document M27-A3.

[13] Espinel-Ingroff A., Turnidge J., Alastruey-Izquierdo A., Botterel F., Canton E., Castro C., Chen Y.-C., Chryssanthou E., Dannaoui E., Garcia-Effron G. (2018). Method-dependent epidemiological cutoff values for detection of triazole resistance in *Candida* and *Aspergillus* species for the Sensititre Yeastone colorimetric broth and Etest agar diffusion methods. Antimicrob. Agents Chemother..

[14] The European Committee on Antimicrobial Susceptibility Testing (2022). Overview of Antifungal ECOFFs and Clinical Breakpoints for Yeasts, Moulds and Dermatophytes Using the EUCAST E.Def 7.3, E.Def 9.4 and E.Def 11.0 Procedures. http://www.eucast.org.

[15] Casalini G., Giacomelli A., Ridolfo A., Gervasoni C., Antinori S. (2021). Invasive fungal infections complicating COVID-19: A narrative review. J. Fungi.

[16] Machado M., Estévez A., Sánchez-Carrillo C., Guinea J., Escribano P., Alonso R., Valerio M., Padilla B., Bouza E., Muñoz P. (2022). Incidence of Candidemia is higher in COVID-19 versus non-COVID-19 patients, but not driven by intrahospital transmission. J. Fungi.

[17] Mastrangelo A., Germinario B.N., Ferrante M., Frangi C., Voti R.L., Muccini C., Ripa M., COVID-BioB Study Group (2020). Candidaemia in coronavirus disease 2019 (COVID-19) Patients: Incidence and characteristics in a prospective cohort compared with historical non–COVID-19 controls. Clin. Infect. Dis..

[18] Cataldo M.A., Tetaj N., Selleri M., Marchioni L., Capone A., Caraffa E., Caro A.D., Petrosillo N., INMICOVID-19 Co-infection Group (2020). Incidence of bacterial and fungal bloodstream infections in COVID-19 patients in intensive care: An alarming “collateral effect”. J. Glob. Antimicrob. Resist..

[19] Pasquini Z., Barocci I., Brescini L., Candelaresi B., Castelletti S., Iencinella V., Mazzanti S., Procaccini G., Orsetti E., Pallotta F. (2021). Bloodstream infections in the COVID-19 era: Results from an Italian multi-centre study. Int. J. Infect. Dis..

[20] McGonagle D., Sharif K., O’Regan A., Bridgewood C. (2020). The Role of Cytokines including Interleukin-6 in COVID-19 induced pneumonia and macrophage activation syndrome-like disease. Autoimmun. Rev..

[21] Fajgenbaum D.C., Khor J.S., Gorzewski A., Tamakloe M.A., Powers V., Kakkis J.J., Repasky M., Taylor A., Beschloss A., Hernandez-Miyares L. (2020). Treatments administered to the first 9152 reported cases of COVID-19: A systematic review. Infect. Dis. Ther..

[22] McCarty T. (2022). Candidemia and Severe Coronavirus Disease 2019: Which Risk Factors Are Modifiable?. Clin. Infect. Dis..

[23] Omrani A.S., Koleri J., Ben Abid F., Daghfel J., Odaippurath T., Peediyakkal M.Z., Baiou A., Sarsak E., Elayana M., Kaleeckal A. (2021). Clinical characteristics and risk factors for COVID-19-associated Candidemia. Med. Mycol..

[24] Kayaaslan B., Kaya Kalem A., Asilturk D., Kaplan B., Dönertas G., Hasanoglu I., Eser F., Korkmazer R., Oktay Z., Ozkocak Turan I. (2022). Incidence and risk factors for COVID-19 associated candidemia (CAC) in ICU patients. Mycoses.

[25] Kundu R., Singla N. (2022). COVID-19 and Plethora of fungal infections. Curr. Fungal Infect. Rep..

[26] Thomas-Rüddel D., Schlattmann P., Pletz M., Kurzai O., Bloos F. (2021). Risk factors for invasive candida infection in critically Ill patients: A systematic review and meta-analysis. Chest.

[27] Dixit D., Jen P., Maxwell T.D., Smoke S., McCracken J.A., Cardinale-King M., Haribhakti A., Patel P., Cani E., Choi S.C. (2022). Risk factors and clinical outcomes of candidemia associated with severe COVID-19. Crit. Care Explor..

[28] Nucci M., Barreiros G., Guimarães L.F., Deriquehem V.A.S., Castiñeiras A.C., Nouér S.A. (2021). Increased incidence of candidaemia in a tertiary care hospital with the COVID-19 pandemic. Mycoses.

[29] Koehler P., Stecher M., Cornely O.A., Koehler D., Vehreschild M.J.G.T., Bohlius J., Wisplinghof H., Vehreschild J.J. (2019). Morbidity and mortality of candidaemia in Europe: An epidemiologic meta-analysis. Clin. Microbiol. Infect..

[30] Fekkar A., Blaize M., Bouglé A., Normand A.C., Raoelina A., Kornblum D., Kamus L., Piarroux R., Imbert S. (2021). Hospital outbreak of fluconazole-resistant *Candida parapsilosis*: Arguments for clonal transmission and longterm persistence. Antimicrob. Agents Chemother..

[31] Castanheira M., Deshpande L.M., Messer S.A., Rhomberg P.R., Pfaller M.A. (2020). Analysis of global antifungal surveillance results reveals predominance of Erg11 Y132F alteration among azole-resistant Candida parapsilosis and *Candida tropicalis* and country-specific isolate dissemination. Int. J. Antimicrob. Agents.

[32] Prigitano A., Cavanna C., Passera M., Gelmi M., Sala E., Ossi C., Grancini A., Calabrò M., Bramati S., Tejada M. (2020). Evolution of fungemia in an Italian region. J. Mycol. Med..

[33] Prigitano A., Cavanna C., Passera M., Ossi C., Sala E., Lombardi G., Grancini A., De Luca C., Bramati S., Gelmi M. (2016). CAND-LO 2014-15 study: Changing epidemiology of candidemia in Lombardy (Italy). Infection.

