# Supplementary material for: Yeast Bloodstream Infections in the COVID-19 Patient: A Multicenter Italian Study (FiCoV Study)
Source: J Fungi (Basel). 2023 Feb 20;9(2):277. doi: 10.3390/jof9020277 (PMC9962415; doi:10.3390/jof9020277)
Supplement: Supplementary file 1 [file jof-09-00277-s001.zip › jof-2113684-supplementary.pdf]

Species (n. isolates tested)	Antifungal agent (n. isolates tested)	No. of isolates with MIC (mg/L) of:															
		0.008	0.015	0.03	0.06	0.125	0.25	0.5	1	2	4	8	16	32	64	128	256
C. albicans (106)	Anidulafungin (86)	-	28	13	21	21			2	1			-	-	-	-	-
	Caspofungin (104)	5	9	25	32	29	2	2					-	-	-	-	-
	Micafungin (85)	32	47	2	1				3				-	-	-	-	-
	Itraconazole (84)	-	6	20	32	23	1		1				1	-	-	-	-
	Fluconazole (104)	-	-	-	-	7	32	52	7	2		1	2			1	
	Voriconazole (104)	53	18	8	2	19	2	1				1	-	-	-	-	-
	Posaconazole (74)	5	22	33	11	1		1				1	-	-	-	-	-
	5-Fluorocytosine (72)	-	-	-	46	22	2	1	1							-	-
	Amphotericin B (95)	-	-	-	-	3	7	60	25				-	-	-	-	-
C. parapsilosis (146)	Anidulafungin (127)		1			1	1	6	92	26		-	-	-	-	-	
	Caspofungin (145)			1		3	14	64	61	2			-	-	-	-	-
	Micafungin (146)	1		1		1	2	27	90	24			-	-	-	-	-
	Itraconazole (125)	-	7	7	14	51	46							-	-	-	-
	Fluconazole (146)	-	-	-	-	3	6	16	13	1		8	6	12	42	37	2
	Voriconazole (145)	5	8	8	2	6	10	50	31	15			-	-	-	-	-
	Posaconazole (120)	4	10	24	51	29	2						-	-	-	-	-
	5-Fluorocytosine (69)	-	-	-	28	32	7	1	1							-	-
	Amphotericin B (140)	-	-	-	-	5	17	94	24				-	-	-	-	-

		0.008	0.015	0.03	0.06	0.125	0.25	0.5	1	2	4	8	16	32	64	128	256
C. glabrata (31)	Anidulafungin (28)		9	12	6	1						-	-	-	-	-	
	Caspofungin (28)		1	4	12	7	4						-	-	-	-	-
	Micafungin (31)		26	5									-	-	-	-	-
	Itraconazole (28)	-				3	1	12	11			1		-	-	-	-
	Fluconazole (28)	-	-	-	-			1		3	7	3	7	4	1	2	
	Voriconazole (27)					4	8	6	9				-	-	-	-	-
	Posaconazole (26)						3	2	9	11	1		-	-	-	-	-
	5-Fluorocytosine (22)	-	-	-	16					2	1	3				-	-
	Amphotericin B (28)	-	-	-	-	1	4	7	16				-	-	-	-	-
C. tropicalis (9)	Anidulafungin (8)		1		1	5	1					-	-	-	-	-	
	Caspofungin (9)			1	4	3	1						-	-	-	-	-
	Micafungin (9)		1	6	2								-	-	-	-	-
	Itraconazole (9)	-		1		1	5		1				1	-	-	-	-
	Fluconazole (9	-	-	-	-			2	1	4	1					1	
	Voriconazole (9)		1		1	2	4		1				-	-	-	-	-
	Posaconazole (7)			1		2	4						-	-	-	-	-
	5-Fluorocytosine (6)	-	-	-	1			1	1	1					2	-	-
	Amphotericin B (8)	-	-	-	-	1	3	4					-	-	-	-	-
Other *Candida* spp.* (4)	Anidulafungin (3)						3					-	-	-	-	-	
	Caspofungin (4)						2	2					-	-	-	-	-
	Micafungin (4)				2	1		1					-	-	-	-	-

		0.008	0.015	0.03	0.06	0.125	0.25	0.5	1	2	4	8	16	32	64	128	256
	Itraconazole (3)	-			2	1								-	-	-	-
	Fluconazole (4)	-	-	-	-		1	1	1	1							
	Voriconazole (4)	2	1			1							-	-	-	-	-
	Posaconazole (3)		1	1	1								-	-	-	-	-
	5-Fluorocytosine (3)	-	-	-	1			1		1						-	-
	Amphotericin B (4)	-	-	-	-		2	1	1				-	-	-	-	-
Saccaromyces (3)	Anidulafungin (3)		1		1							-	-	-	-	-	
	Caspofungin (3)			1	2								-	-	-	-	-
	Micafungin (3)				1	2							-	-	-	-	-
	Itraconazole (3)	-							2				1	-	-	-	-
	Fluconazole (3)	-	-	-	-	2	1										
	Voriconazole (3)						1	2					-	-	-	-	-
	Posaconazole (3)								1	2			-	-	-	-	-
	5-Fluorocytosine (3)	-	-	-	2								1			-	-
	Amphotericin B (3)	-	-	-	-	2	1						-	-	-	-	-

Table S1. In vitro susceptibilities of 299 bloodstream isolates.

- Concentrations not investigated

* Other *Candida* species include *C. lusitaniae* (2) and *C. metapsilosis* (2).
